# Using the geometric average hazard ratio in sample size calculation for time-to-event data with composite endpoints

**DOI:** 10.1186/s12874-021-01286-x

**Published:** 2021-05-06

**Authors:** Jordi Cortés Martínez, Ronald B. Geskus, KyungMann Kim, Guadalupe Gómez Melis

**Affiliations:** 1 Department of Statistics and Operations Research, Universitat Politècnica de Catalunya, Jordi Girona, 31, Barcelona, 08034 Spain; 2 Oxford University Clinical Research Unit, Ho Chi Minh City, Vietnam; 3 Centre for Tropical Medicine and Global Health, Nuffield Department of Medicine, Oxford, United Kingdom; 4 Department of Biostatistics and Medical Informatics, University of Wisconsin, 600 Highland Ave, Madison, 53792 USA

**Keywords:** Treatment Effect, Composite endpoint, Randomized Controlled Trial, Progression-Free Survival, Simulation, Copula, Non-Proportional Hazards

## Abstract

**Background:**

Sample size calculation is a key point in the design of a randomized controlled trial. With time-to-event outcomes, it’s often based on the logrank test. We provide a sample size calculation method for a composite endpoint (CE) based on the geometric average hazard ratio (*gAHR*) in case the proportional hazards assumption can be assumed to hold for the components, but not for the CE.

**Methods:**

The required number of events, sample size and power formulae are based on the non-centrality parameter of the logrank test under the alternative hypothesis which is a function of the *gAHR*. We use the web platform, CompARE, for the sample size computations. A simulation study evaluates the empirical power of the logrank test for the CE based on the sample size in terms of the gAHR. We consider different values of the component hazard ratios, the probabilities of observing the events in the control group and the degrees of association between the components. We illustrate the sample size computations using two published randomized controlled trials. Their primary CEs are, respectively, progression-free survival (time to progression of disease or death) and the composite of bacteriologically confirmed treatment failure or Staphylococcus aureus related death by 12 weeks.

**Results:**

For a target power of 0.80, the simulation study provided mean (± SE) empirical powers equal to 0.799 (±0.004) and 0.798 (±0.004) in the exponential and non-exponential settings, respectively. The power was attained in more than 95% of the simulated scenarios and was always above 0.78, regardless of compliance with the proportional-hazard assumption.

**Conclusions:**

The geometric average hazard ratio as an effect measure for a composite endpoint has a meaningful interpretation in the case of non-proportional hazards. Furthermore it is the natural effect measure when using the logrank test to compare the hazard rates of two groups and should be used instead of the standard hazard ratio.

## Background

Composite endpoints (CEs), defined as the union of several outcomes, are extensively used as a primary endpoint when designing a clinical trial. In time-to-event studies, CE refers to the elapsed time from randomization to the earliest observation among its components. It is common in oncology trials to use progression-free survival (PFS) as a primary endpoint: this outcome is defined as the time elapsed from randomization to tumor progression or death from any cause, whichever occurs first [1]. In cardiovascular trials, major adverse cardiac event (MACE) is generally defined as a composite endpoint of time to cardiovascular death, myocardial infarction, stroke and target vessel revascularization [2]. Composite endpoints are as well often used for infectious diseases. In the ARREST trial [3], the primary endpoint was the time to bacteriologically confirmed treatment failure, disease recurrence or death.

In randomized controlled trials (RCTs), to assess the efficacy of an intervention on a time-to-event endpoint, the hazard ratio (HR) is routinely used. Design and analysis in most RCTs are based on the proportional hazards model, even when this proportionality is not met. Royston et al. [4] explored 55 comparisons in 50 published RCT and found evidence of non-PH at the 0.10 level almost in 1 out of 3 comparisons (31%) that had assumed proportional hazards for the sample size calculations. This is often the case in RCT with a CE even if the proportionality assumption holds for each component endpoint.

The conventional formulae for the required number of events with regard to the defined primary endpoint in time-to-event studies depends, given the specified significance level and power, on a single treatment effect summarized by the hazard ratio anticipated under the alternative hypothesis. The number of patients that have to be recruited to observe the calculated number of events depends on, among others, the probability of observing the event during the follow-up. In the context of a trial with a CE, if these formulae are to be used, it is necessary to decide on a summary for the hazard ratio of the CE, *HR*_∗_(*t*).

Different summaries for HR_∗_(*t*) have been put forward such as the average hazard ratio (AHR) proposed by Kalbfleisch and Prentice [5], and the geometric average hazard ratio (gAHR). Schemper et al. [6] compares these average hazard ratios, and explores weighted versions of *AHR* and *gAHR*. While under proportional hazards all definitions lead to the same values, under non-proportional hazards both the unweighted and the weighted versions of AHR and gAHR are close to each other except when the hazards cross. We emphasize here the use of the gAHR as it nicely connects with the logrank test, as opposed to the AHR. Our paper has some analogy with Rauch et al.’s work [7], who provide guidance on the practical use of the average hazard ratio introduced by Kalbfleisch and Prentice [5].

This paper focuses on the sample size calculation for a clinical trial with a primary composite endpoint. We start by introducing the notation, some of the assumptions and definitions. Next, the logrank test and the noncentrality parameter is set forth and based on the connection between the two, the number of events and sample size formulae for a two-sample problem based on a two-component composite endpoint (CE) are provided. Following this, software CompARE (https://cinna.upc.edu/CompARETimeToEvent/) is introduced and we show how to use it to design trials with composite endpoints in the setting in which each composite endpoint approximately satisfies the proportionality hazard assumption. Its application is illustrated by means of two real RCTs, ZODIAC and ARREST. Then, the results of a simulation study of the empirical power based on the sample size formula previously derived are shown. These simulations are run for several scenarios including different values of the component cause-specific hazard ratios, a wide range of probabilities of observing the events in the control group and different degrees of association between the components. We conclude the paper with a discussion.

## Methods

### Geometric average hazard ratio

In such an RCT, individuals are followed until the event of interest (${\mathcal {E}}_{1}$ or ${\mathcal {E}}_{2}$), the end of the study or censoring due to random loss to follow-up. For each group *g* (*g*=0,1) we denote by $T^{(g)}_{1}$ and $T^{(g)}_{2}$ the times to ${\mathcal {E}}_{1}$ and ${\mathcal {E}}_{2}$, respectively, and by $T^{(g)}_{*}$ the time to the occurrence of ${\mathcal {E}}_{*} $, i.e., the earlier occurrence of ${\mathcal {E}}_{1}$ or ${\mathcal {E}}_{2}$. We are in this case dealing with a competing risk situation for which several approaches are possible, one well known alternative being the Fine and Gray model. However, we have chosen to model the cause-specific hazards because it can be accomplished with standard methods for single types of events by treating all competing events as right censored at the time the competing event occurs [8]. In addition, most often the cause-specific hazard is reported from previous studies that provide the basis for the assumed effect size If we view the events ${\mathcal {E}}_{1}$ and ${\mathcal {E}}_{2}$ as the two causes of the composite event ${\mathcal {E}}_{*}$, then for *g*=0,1, the cause-specific hazard rates, $\lambda ^{(g)}_{C1}(t)$ and $\lambda ^{(g)}_{C2}(t)$, the all-cause hazard rate corresponding to the hazard of the composite endpoint $T^{(g)}_{*}, \lambda _{*}^{(g)}(t)$, and the survival function of $T^{(g)}_{*}, S^{(g)}_{*}(t)$, are expressed, respectively, as

$$\begin{array}{@{}rcl@{}} \lambda^{(g)}_{C1}(t)&=&{\lim}_{\Delta t\rightarrow 0^{+}}\text{Prob}\left\{t\leq T^{(g)}_{1}< t+\Delta t, T^{(g)}_{1}\right.\\&&\qquad\qquad\qquad\left.<T^{(g)}_{2} |T^{(g)}_{*}\geq t\right\}/{\Delta t}\\ \lambda^{(g)}_{C2}(t)&=&{\lim}_{\Delta t\rightarrow 0^{+}}\text{Prob}\left\{t\leq T^{(g)}_{2}< t+\Delta t, T^{(g)}_{2}\right.\\&&\qquad\qquad\qquad\left.<T^{(g)}_{1} |T^{(g)}_{*}\geq t\right\}/{\Delta t}\\ \lambda_{*}^{(g)}(t)\!&=&\! \lambda^{(g)}_{C1}(t)\,+\, \lambda^{(g)}_{C2}(t); \quad S^{(g)}_{*}(t)=e^{-\int_{0}^{t}\left(\lambda_{C1}^{(g)}(u)+\lambda_{C2}^{(g)}(u)\right)du} \end{array} $$

Denote by HR_*k*_(*t*) (*k*=1,2) the cause-specific hazard ratios, that is, the hazard ratios of the individual components, and by HR_∗_(*t*) the all-cause hazard ratio of the composite endpoint $T^{(g)}_{*}$.

Define the geometric average hazard ratio, gAHR, as the exponentiated mean of the logarithm of the hazard ratio, that is,

1 $$  \text{gAHR}=\exp\left\{\mathrm{E}(\log \text{HR}_{*}(T)) \right\}  $$

where the expectation is taken with respect to a given event-time distribution, which in this case is the average distribution of $T_{*}^{(0)}$ and $T_{*}^{(1)}$ (see Eq. 2 below). Although *HR*_∗_(*t*) is expected to change over time, *gAHR* is independent of time, keeps its interpretability under non proportional hazards and, as we will see in “Logrank test for the composite endpoint *T*_∗_” section, is the natural effect measure when using the logrank test.

Because within a clinical trial there is a maximum follow-up time, say *τ*, only a restricted version of the geometric average hazard ratio can be consistently estimated. Define the truncated geometric average hazard ratio at time *τ*,*gAHR*(*τ*) as

2 $$\begin{array}{@{}rcl@{}}  gAHR(\tau)&=&\exp\left\{\frac{\int_{0}^{\tau} \log\left\{\frac{\lambda_{*}^{(1)}(t)}{\lambda_{*}^{(0)}(t)} \right\} f^{(a)}_{*}(t) dt} {\int_{0}^{\tau} f^{(a)}_{*}(t) dt} \right\}\\ &=&\exp\left\{\frac{\int_{0}^{\tau} \log\left\{\frac{\lambda_{*}^{(1)}(t)}{\lambda_{*}^{(0)}(t)} \right\} f^{(a)}_{*}(t) dt} {p^{(a)}_{*}(\tau)} \right\} \end{array} $$

where $f^{(a)}_{*}(t)=(f_{*}^{(0)}(t)+f_{*}^{(1)}(t))/2$ is the average of the density functions of $T_{*}^{(0)}$ and $T_{*}^{(1)}, f^{(g)}_{*}(t)= -\frac { \partial }{\partial t} S^{(g)}_{*}(t)$ is the density function of $T^{(g)}_{*} $ (*g*=0,1) and $p^{(a)}_{*}(\tau)=\int _{0}^{\tau } f^{(a)}_{*}(t) dt =(p^{(0)}_{*}(\tau)+p^{(1)}_{*}(\tau))/2$ is the average probability of experiencing the event ${\mathcal {E}}_{*}$ over both groups by time *τ*.

The geometric average hazard ratio and the all-cause hazard ratios take identical values under proportionality of the all-cause hazard rates, that is, if $HR_{*}(t)=\lambda _{*}^{(1)}(t)/\lambda _{*}^{(0)}(t)=h$ for 0<*t*<*τ*, then *gAHR*(*τ*)=*HR*_∗_(*t*)=*h*, for 0<*t*<*τ*.

### Logrank test for the composite endpoint *T*_∗_

The hypothesis of no treatment difference when we are using the composite endpoint *T*_∗_ is stated in terms of the all-cause hazard rates for *T*_∗_, that is, $H_{0}^{*}\!: \lambda _{*}^{(0)}(\cdot)=\lambda _{*}^{(1)}(\cdot)$.

The standard approach to assess the above comparison is the logrank test. Assume that we have *n* patients, with *n*^(0)^=(1−*π*)·*n* allocated to treatment group 0 and *n*^(1)^=*π*·*n* to group 1, where *π* is the proportion of individuals allocated to group 1. Denoting by *d*_∗_ the total number of patients from both groups who have experienced event ${\mathcal {E}}_{*}$ (either ${\mathcal {E}}_{1}$ or ${\mathcal {E}}_{2}$), say at times *t*_*i*_ (*i*=1,⋯,*d*_∗_) and by *R*^(*g*)^(*t*_*i*_) the number of individuals at risk at time *t*_*i*_ from group *g* (*g*=0,1), the logrank test statistic *Z*_∗_ can be expressed as

3 $$ {}Z_{*}\,=\, \frac{\sum_{i=1}^{d_{*}}\!\left(\!\mathbf{1}\{\text{event\ at \ time}\ t_{i} \text{ is\ in\ group\ 1}\}\,-\,\frac{R^{(1)}(t_{i})}{R^{(0)}(t_{i})+R^{(1)}(t_{i})}\!\right)}{\sqrt{\sum_{i=1}^{d_{*}}\frac{R^{(0)}(t_{i})R^{(1)}(t_{i})}{(R^{(0)}(t_{i})+R^{(1)}(t_{i}))^{2}}}}  $$

and one rejects $H_{0}^{*}$ when |*Z*_∗_| is large.

The large sample behaviour of *Z*_∗_ is studied by Schoenfeld [9] who shows that *Z*_∗_, under the null hypothesis of equality of the survival distributions in the two groups, is asymptotically normal with mean 0 and unit variance. Since for any fixed alternative to $H_{0}^{*}$, the power of *Z*_∗_ will typically go to 1 as *n*→*∞*, the large sample behaviour of *Z*_∗_ when $H_{0}^{*}$ does not hold is studied for a sequence of contiguous alternatives to $H_{0}^{*}$ which approach $H_{0}^{*}$ as *n*→*∞*. That is, we view $\lambda _{*}^{(0)}(\cdot)$ as fixed, let $\lambda _{*}^{(1)}(\cdot)$ vary with *n* and define the sequence of contiguous alternatives to $H_{0}^{*}$ as $H^{*}_{a,n}\!:\lambda ^{(1)}_{*,n}(t)=\lambda _{*}^{(0)}(t)e^{g(t)/\sqrt n},$ stating that for any finite *n*, the two groups have a log hazard ratio at time *t* equal to $g(t)/\sqrt n$. Under these conditions *Z*_∗_ is also approximately unit-variance normal, but with a non-zero mean that depends on the survival and censoring distributions in the two groups, and the proportion of subjects that are in each group. The asymptotic theory behind these results is analogous to what is done when studying the large-sample properties of likelihood-based tests in more standard settings (where there is no censoring). The reader is referred to Section 3.3 in Lehmann [10] for additional technical details about the contiguous alternative hypotheses.

Under any form of *g*(*t*), even constant, Gómez and Lagakos [11] applied this result for a composite endpoint under the assumption of non-informative censoring. They showed that for a sufficiently large time, *τ*, the noncentrality parameter *μ*_∗_ is, approximately, as follows

4 $$\begin{array}{@{}rcl@{}} \mu_{*} (\tau)&=&\frac{\sqrt{n\pi(1-\pi)} \int_{0}^{\tau} \log\left\{\frac{\lambda^{(1)}_{*,n}(t)}{\lambda^{(0)}_{*}(t)}\right\} f_{*}^{(0)}(t) dt}{\sqrt{ p_{*}^{(0)}(\tau) }} \end{array} $$

where $ f^{(0)}_{*}(t)$ is the marginal density function for $T^{(0)}_{*}$.

This allows evaluation of the behaviour of the logrank test under alternatives where the hazard functions for the two groups are non-proportional, as is the case in the composite endpoints situation that we are dealing with. Furthermore, if we replace $ f^{(0)}_{*}(t)$ by the average of the density functions of $T_{*}^{(0)}$ and $T_{*}^{(1)}, f^{(a)}_{*}(t)$, which trivially equals to $ f^{(0)}_{*}(t)$ under $H_{0}^{*}$, the expression (4) for the noncentrality parameter, becomes

5 $$\begin{array}{@{}rcl@{}} \mu_{*} (\tau)&=&\frac{\sqrt{n\pi(1-\pi)} \int_{0}^{\tau} \log\left\{\frac{\lambda_{*}^{(1)}(t)}{\lambda_{*}^{(0)}(t)} \right\} f_{*}^{(a)}(t) dt}{\sqrt{ p_{*}^{(a)}(\tau) }} \end{array} $$

or equivalently, using the expression for *gAHR*(*τ*) in (2),

6 $$\begin{array}{@{}rcl@{}} \mu_{*}(\tau) &=&\sqrt{n\pi(1-\pi) p_{*}^{(a)}(\tau)} \log({gAHR(\tau)}), \end{array} $$

showing that it depends on the geometric average hazard ratio without relying either on the proportionality of the cause-specific hazard rates or on the all-cause hazard rates.

### Sample size estimation

Assume that you are planning a RCT based on a composite endpoint as the primary endpoint, that you are basing the comparison between the two groups on the logrank test statistic *Z*_∗_ given in (3) and that the geometric average hazard ratio is used as a measure of the treatment effect. From now on, the focus is to claim superiority of the new therapy (*g*=1), hence, the logrank test statistic *Z*_∗_ given in (3) will be used and the null hypothesis will be rejected for a one-sided *α* significance level whenever *Z*_∗_<−*z*_*α*_ where *z*_*α*_ is the *α*-quantile of the standard normal distribution, noting that negative values of *Z*_∗_ favor the new therapy.

The asymptotic results in previous subsection may be applied to a fixed sample size *n* and a fixed alternative, hence the expression (6) of *μ*_∗_(*τ*) can be used to plan the size, power and the duration of a study. Using that *Z*_∗_, follows a normal distribution with mean *μ*_∗_(*τ*) and variance 1, the power 1−*β* is such that

7 $$\begin{array}{@{}rcl@{}} 1-\beta&=&\text{Prob}\{Z_{*}<-z_{\alpha}\}, \end{array} $$

it follows from (7) that −*z*_*α*_−*μ*_∗_(*τ*)=−*z*_1−*β*_=*z*_*β*_⇒*μ*_∗_(*τ*)=−(*z*_*α*_+*z*_*β*_), and equating with (6) we have

8 $$ {} \mu_{*} (\tau)=\sqrt{n\pi(1-\pi) p_{*}^{(a)}(\tau)} \log(gAHR(\tau))=-(z_{\alpha}+z_{\beta}).  $$

The total sample size for both groups is therefore as follows:

9 $$\begin{array}{@{}rcl@{}} n&=&\frac{(z_{\alpha}+z_{\beta})^{2}}{\pi(1-\pi)p_{*}^{(a)}(\tau) \left(\log(gAHR(\tau)\right)^{2}} \end{array} $$

and the expected number of CE events $e_{*}=n\cdot p_{*}^{(a)}(\tau)$ is given by

10 $$\begin{array}{@{}rcl@{}} e_{*}&=&\frac{(z_{\alpha}+z_{\beta})^{2}}{\pi(1-\pi) \left(\log(gAHR(\tau)\right)^{2}} \end{array} $$

In the special case of equal sample sizes (*π*=0.5), (9) and (10) become, respectively,

11 $$\begin{array}{@{}rcl@{}} n&=&\frac{4(z_{\alpha}+z_{\beta})^{2}}{p_{*}^{(a)}(\tau) \left(\log(gAHR(\tau)\right)^{2}} \end{array} $$

12 $$\begin{array}{@{}rcl@{}} e_{*}&=&\frac{4(z_{\alpha}+z_{\beta})^{2}}{\left(\log(gAHR(\tau)\right)^{2}}  \end{array} $$

To obtain expression (12) from expression (11) (or vice versa), the same follow-up period (*τ*) had to be assumed for all study participants, regardless of how recruitment was carried out. Although we consider this to be a common approach in clinical trials, it is not the only one. Another option would be for recruitment to take place over a certain duration and for subsequent follow-up to be done up to a fixed point in time. These and other strategies are well explained in Chapter 8 of the book of Friedman et al. [12]. Nevertheless, the key point is in the estimation of the number required of events because that is where a difference may be compared to other methods. Once the number of events required is estimated, the effect of the recruitment rate or the presence of different follow-up periods on sample size calculation — i.e. the number of patients required — would be the same across different methods. There are several references that deal with this issue. The article of Lachin et al. [13] shows how to go from the number of events to the number of patients under situations where patients enter the trial in a nonuniform manner over time or patients may exit from the trial due to loss to follow-up. Besides, Bernstein et al. [14] provide a routine in Fortran to estimate the probabilities of observing the event from the parameters referring to recruitment and follow-up times.

Observe that, formula (12) corresponds to George and Desu’s formula [15] (known these days as Schoenfeld’s formula [9]) for the required number of events if the hazard ratio is substituted by the geometric average hazard ratio. The main difference lies, however, in that while in Schoenfeld’s formula you are assuming that the hazard rates are proportional when dealing with a composite endpoint the all-cause hazard rates do not have to be proportional. However, to be able to compute the *gAHR*(*τ*) you need extra distributional assumptions that are described in the next section.

### CompARE: a software application to design trials with composite endpoints

We introduce here the web-based application CompARE (https://cinna.upc.edu/CompARETimeToEvent/) that will be used for the sample size computations. CompARE is a website specifically created to design clinical trials with composite endpoints. From input parameters such as the cause-specific hazard ratios, the probabilities of observing each event, the shape parameters of the distributions of the time to each component (assumed Weibull) and the correlation between marginal distributions, CompARE computes and plots, among others, the hazard ratio along time, *HR*_∗_(*t*), summaries such as the geometric average hazard ratio, *gAHR*(*τ*), and the restricted mean survival time, *RMST*(*τ*), and calculates the sample size for a given significance level and power.

CompARE has been built for balanced designs (equal sample size in both groups) and depends on anticipated values provided by the users. In particular, in order to compute the required sample size by means of (11) we need to compute *gAHR*(*τ*) and $p_{*}^{(a)}(\tau)$ and to do so we have to make distributional assumptions and provide the values of several parameters. Specifically we need, for each group (*g*=0,1), the distribution of the composite endpoint $T^{(g)}_{*}=\min \{T^{(g)}_{1}, T^{(g)}_{2}\}$, which is derived from the joint distribution between $T^{(g)}_{1}$ and $T^{(g)}_{2}$. In what follows we itemize the elements implemented in CompARE for a full characterization of the joint distribution.

1. The joint distribution between $T^{(g)}_{1}$ and $T^{(g)}_{2}$ is modeled through a copula. The copula binds the marginal distributions of $T^{(g)}_{1}$ and $T^{(g)}_{2}$ through an association parameter. CompARE can use several copulas, but in this work we use Archimedean copulas by Frank, Clayton, and Gumbel (see [16]) as the more appropriate for modeling event-time data, providing different dependence characteristics.The measure of association between $T_{1}^{(g)}$ and $T_{2}^{(g)}$ is given by Spearman’s rank correlation coefficient *ρ* or Kendall’s *τ*. We assume that these measures of association are the same in both groups. If *C*(*u*,*v*;*θ*) denotes the chosen copula and *θ* is the association parameter, *τ* and *ρ* are defined as follows:
  13 $$\begin{array}{@{}rcl@{}} \rho&=&12\int_{0}^{1}\int_{0}^{1}[C(u,v;\theta)-uv]dudv \\ \tau&=&4\int_{0}^{1}\int_{0}^{1}C(u,v; \theta)dC(u,v; \theta)-1 \end{array} $$
2. The marginal laws for $T^{(g)}_{k}$ (*g*=0,1;*k*=1,2) are from the Weibull family of distributions. The Weibull law depends on a scale and a shape parameter. It has been chosen because it is flexible enough to represent different life-time data scenarios, allowing increasing, constant (exponential model) and decreasing hazard functions, although would not be valid for non-monotonous hazard functions. Furthermore Weibull distributions for both groups result in proportional hazards if they share the same shape parameter. The exponential law, which is often the preferred choice for sample size calculations, is a special case when the shape parameter equals to 1. While the shape parameters (*β*_1_,*β*_2_) of the Weibull distributions are given as inputs by the researcher, the scale parameters $\left (b_{1}^{(0)}, b_{2}^{(0)},b_{1}^{(1)}, b_{2}^{(1)}\right)$ are determined via:
  - The probabilities $p_{1}^{(0)}=p_{1}^{(0)}(\tau)$ and $p_{2}^{(0)}=p_{2}^{(0)}(\tau)$ of observing endpoints $T_{1}^{(0)}$ and $T_{2}^{(0)}$. They are defined, taking into account the competing risk setting, as $p^{(0)}_{1}=\text {Prob}\left \{T^{(0)}_{1}<\tau, T^{(0)}_{1}<T^{(0)}_{2}\right \}$ and $p^{(0)}_{2}=\text {Prob}\left \{T^{(0)}_{2}<\tau, T^{(0)}_{2}<T^{(0)}_{1}\right \}$ based on the joint distribution of $T^{(g)}_{1}$ and $T^{(g)}_{2}$, which has been modeled through the copula *C*(*u*,*v*;*θ*) explained in item 1. In those cases when ${\mathcal {E}}_{2}$ (analogously for ${\mathcal {E}}_{1}$) does not represent a fatal event, then we can observe all the occurrences of ${\mathcal {E}}_{1}$ and define $p^{(0)}_{1}=\text {Prob}\{T^{(0)}_{1}<\tau \}$. The scale parameters in the reference group (*g*=0) are function of the joint density $f_{(1,2)}^{(0)}(\cdot,\cdot ;\theta)$ and are computed as the solution of the following equations:
    14 $$\begin{array}{@{}rcl@{}} p_{1}^{(0)}=\int_{0}^{\tau}\left(\int_{u}^{\tau}f_{(1,2)}^{(0)}(u, v;\theta) dv\right)du \\ p_{2}^{(0)}=\int_{0}^{\tau}\left(\int_{v}^{\tau}f_{(1,2)}^{(0)}(u, v;\theta) du\right)dv \end{array} $$
  - The cause-specific hazard rates $\lambda ^{(g)}_{C1}(t)$ and $\lambda ^{(g)}_{C2}(t)$ (*g*=0,1) for ${\mathcal {E}}_{1}$ and ${\mathcal {E}}_{2} $, respectively. We assume that treatment groups have proportional cause-specific hazard rates for each component and denote by HR_1_ and HR_2_ the respective cause-specific hazard ratios, that is,
    15 $$\begin{array}{@{}rcl@{}} {}\text{HR}_{1}&=&\lambda_{C1}^{(1)}(t)/\lambda_{C1}^{(0)}(t)\quad \text{for\ all} \quad t<\tau  \\ {}\text{HR}_{2}&=&\lambda_{C2}^{(1)}(t)/\lambda_{C2}^{(0)}(t) \quad \text{for\ all} \quad t<\tau \end{array} $$
    Without loss of generality assume that both events ${\mathcal {E}}_{1}$ and ${\mathcal {E}}_{2}$ are undesirable and that the new therapy is expected to reduce the risk of both events, that is, *HR*_*k*_<1,*k*=1,2. The proportionality of the cause-specific hazard ratios HR_1_ and HR_2_ allows us to compute the scale parameters in the new therapy group (g=1). For more details, the reader is refered to the supplementary material in Gómez and Lagakos [11] for the relationship between the scale parameters with $\left (p_{1}^{(0)}, p_{2}^{(0)}\right)$ and (HR_1_,HR_2_).

In many phase III RCTs the results from earlier trials provide the rationale for the further assessment of the intervention, and could be used for the sample size calculation. In other situations, similar clinical trials or observational studies could be used. However, as in most sample size settings, obtaining the required parameters is not straightforward and the resulting sample size may depend heavily on the appropriate choice of those. In the composite endpoint setting the choice of the copula and the association between the marginals adds complexity. Since CompARE allows the users to compute the sample size under different scenarios, the influence on the sample size of the different choices can be studied, providing a basis for a more informed decision.

## Results

### Case studies

#### The ZODIAC trial

We illustrate the technique for sample size determination using the ZODIAC trial [17]. The trial compared the efficacy of vandetanib plus docetaxel versus placebo plus docetaxel as second-line treatment in patients with advanced non-small-cell lung cancer. The co-primary endpoints of this trial were overall survival (OS) and progression free survival (PFS), defined as the absence of death and disease progression (DP).

A total of 1,176 events (both groups) were determined to be necessary to detect at least a 20% reduction (*HR*<0.8) on PFS treatment effect using a two-tailed logrank test with 0.90 power and 0.0242 level of significance (they adjusted for multiplicity to simultaneously assess both co-primary endpoints). They enrolled 1,391 patients which were followed between 3 to 24 months. The trial was conducted between May 2006 and April 2008 and, at the end of the study, the reported cause-specific HRs for each component were 0.91 (OS) and 0.77 (time to progression, TTP) and the estimated HR of the composite endpoint, PFS, was 0.79. The probabilities of observing DP or deaths (accounting for those subsequent to the DP) were, respectively, 0.74 and 0.59. They concluded that vandetanib in combination with docetaxel significantly improves PFS compared with placebo plus docetaxel.

Assume that a future trial for advanced non-small-cell lung cancer is to be conducted using PFS as the composite primary endpoint and aiming to prove the effect of an intervention on the PFS through the geometric hazard ratio *gAHR*. We can use the abovementioned reported values in the ZODIAC trial as our anticipated parameters, allow for different magnitudes for the association between death and TTP and for different patterns (constant, decreasing or increasing) for the cause-specific hazard rates. A total of 5 scenarios have been considered. In all but the first, a moderate correlation has been assumed (Spearman’s *ρ*=0.5). The first two scenarios follow the classic assumption in sample size calculations of exponentiality (Weibull shape parameters *β*_1_=*β*_2_=1), the first assuming weak correlation between OS and TTP (Spearman’s *ρ*=0.1). Scenarios 3 and 4 assume exponentiality (*β*_1_=1) for OS but increasing (*β*_2_=2) and decreasing (*β*_2_=0.5) hazard rates over time for TTP, respectively. Scenario 5 assumes increasing hazard rate (*β*_1_=2) for OS and decreasing (*β*_2_=0.5) for TTP. Scale parameters were worked out from the reported cause specific hazard ratios and the probabilities of observing DP ($p_{2}^{(0)}=0.74$) and death ($p_{1}^{(0)}=0.59$, including those after DP) (see item 2a in previous section). Based on ZODIAC’s trial follow-up, we set a fixed *τ*=24 months.

Table 1 summarizes the results for the 5 scenarios. CompARE provided the geometric average hazard ratio evaluated at 24 months, *gAHR*(24), the required number of events to achieve 90% power using Eq. 12, the probability of observing the composite event in either group, $p_{*}^{(a)}$ and the corresponding sample size using Eq. 11. The empirical power with this sample size was obtained via simulation with 10,000 runs.

**Table 1:** Summary characteristics of the five scenarios considered to emulate the ZODIAC trial

	*β*_1_	*β*_2_	*ρ*	gAHR(24)	Events	${p_{*}^{(a)}}$	N	Emp. Power
Scenario 1	1	1	0.1	0.808	1,106	0.952	1,162	0.894
Scenario 2	1	1	0.5	0.816	1,208	0.900	1,344	0.898
Scenario 3	1	2	0.5	0.803	1,044	0.981	1,066	0.896
Scenario 4	1	0.5	0.5	0.823	1,313	0.842	1,560	0.895
Scenario 5	2	0.5	0.5	0.825	1,349	0.814	1,658	0.902

***gAHR(24)***: geometric Average Hazard Ratio at 24 months; $\boldsymbol {p}_{\mathbf {*} }^{\boldsymbol {(a)}}$: Probability of observing composite event in either group; ***N***: Sample size (both groups combined) rounded to an even number

The geometric average hazard ratio evaluated at 24 months, *gAHR*(24), slightly changes among scenarios, ranging from 0.803 to 0.825, and is close both to the reported point estimate of PFS hazard ratio (0.79) and to the PFS hazard ratio used in the sample size calculation (0.80). These small differences in the effect measure cause a reduction of 305 events when going from Scenario 5 to Scenario 3. This reduction together with a very large probability of observing the composite event in Scenario 3 implies almost 600 fewer patients in the total sample size for this scenario.

We can also observe that the monotonicity pattern of the cause-specific hazard rates, determined by the shape parameters *β*_*j*_, has an important influence on the probability of observing the composite event and, consequently, on the sample size. Finally, the degree of association has as well some impact on the required number of events. For instance, when comparing Scenarios 1 and 2, we need 102 extra events in the later due to a higher association between OS and TTP.

After running simulations with these scenarios, the obtained empirical powers were very close to the target power (0.9) and they do not seem to be influenced neither by different hazard behaviors nor by the association magnitude. We conducted simulations under other settings (not shown) with other possible combinations of *β*_1_ and *β*_2_ (*β*_*j*_∈{0.5,1,2}) and for a wide spectrum of correlations (*ρ*∈{0.1,0.3,0.5,0.8}) and the minimum empirical power achieved was 0.893.

**Fig. 1:**
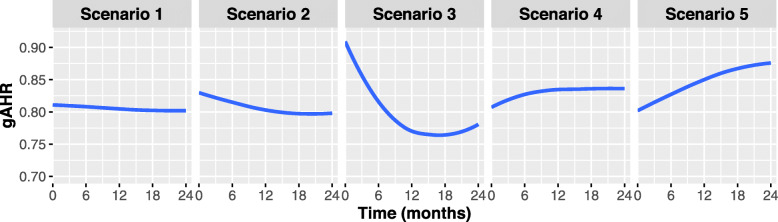
ZODIAC trial. HR_∗_(*t*) over time in 5 plausible scenarios for the ZODIAC trial: 1) weak correlation (*ρ*=0.1) and constant hazards for OS and TTP; The remaining scenarios have moderate correlation (*ρ*=0.5) but with different hazard behavior for OS and TTP: 2) both constant (OS and TTP); 3) constant (OS) and increasing (TTP); 4) constant (OS) and decreasing (TTP); and 5) increasing (OS) and decreasing (TTP)

Finally, we explored the influence that different marginal hazard rate patterns could have on the behaviour of the PFS hazard ratio, *HR*_∗_(*t*), and Fig. 1, reproduced with CompARE, depicts them. While in the first two scenarios, where both components are exponentially distributed, *HR*_∗_(*t*) does not vary much over time and a summary measure such as the hazard ratio could capture well enough the effect of the intervention, in the remainder three scenarios *HR*_∗_(*t*) could vary between 0.77 and 0.90. The first 3 scenarios would correspond to interventions with a decrease in hazard ratio over time, while in the last two, the efficacy would be greater at the beginning of the follow-up. These graphs show the relevance of the behavior of the hazard rates in the evolution of treatment efficacy over time.

#### The ARREST trial

The ARREST trial [3] was the first large randomized clinical trial of antibiotic therapy in patients with *Staphylococcus aureus (SA)* bloodstream infection. It tested the hypothesis that adjunctive rifampicin improves disease outcome. The study was designed with a composite primary outcome: bacteriologically confirmed treatment failure or disease recurrence or death by week 12.

Assuming 0.80 power and a two-sided test with size *α*=0.05, 770 participants were needed to detect a 30% relative reduction (from 35% to 25%) in the composite primary endpoint. In addition, a decrement in the percentage of deaths from 16 to 9% was anticipated. A 10% and 4% losses to follow-up were assumed in each component, respectively. No differences were found in the primary composite endpoint, HR_∗_=0.96 (95% CI, from 0.68 to 1.35). The HR for overall survival was HR_*OS*_=1.10 (95% CI, from 0.76 to 1.60), leading to a point estimate of the overall survival treatment effect in the opposite direction than the one expected.

This new case study has interest on its own because of the following differences with the ZODIAC trial. First, the ARREST trial includes three outcomes and shows that having more than two outcomes of interest does not limit our methodology. In this case we can combine several outcomes into a single component, as long as we can anticipate the parameters required in the calculation of the sample size for such components and the new HR is reasonably constant. Second, there is a huge difference in the proportion of events; while in the ZODIAC trial, more than 50% of the patients suffered any event, in the ARREST trial, none of the involved events was present in more than 15% of the patients. Third, since we have had access to the ARREST raw data, this illustration shows how a previous study might help to set the input parameters for the sample size calculation.

Suppose we want to carry out a new RCT to show the efficacy of adjunctive rifampicin in reducing bacteriologically confirmed treatment failure, disease recurrence or death by week 12. These three outcomes will be considered and their composite will be chosen as the primary endpoint. Overall survival is our first component, while non-fatal events (bacteriologically confirmed treatment failure or disease recurrence) will conform the second component, which in fact is the most relevant because it more closely reflects the treatment effect. Only for the purpose of this illustration, we are assuming a weak beneficial treatment effect on the all-cause mortality (HR_1_=0.95) and a large effect on the bacteriologically confirmed treatment failure or disease recurrence (HR_2_=0.35). The probabilities of observing each component event in the control group were 0.14 and 0.05 for fatal and non-fatal events, respectively. In the competing-risks setting, like this one, marginal time-to-event distributions are not directly estimable from the raw data [18]. However, at the design stage, we have to anticipate the shape parameters. On one hand, the OS shape parameter can be estimated because we have the information on all deaths (before and after the non-fatal events), obtaining $\beta _{1}^{(0)}=0.7$. We are aware that when considering deaths after PD, we are not dealing with the marginal distribution in a context of competing risks, but we consider this a good approximation. For the non-fatal events, and since we have partial information from the trial, we have computed the shape parameter $\beta _{2}^{(0)}$ for different potential scenarios, obtaining values that range from 0.9 to 2.7. These different values lead to sample sizes ranging from 3,136 to 3,350. Only for the purpose of this illustration, we have assumed that the non-fatal events are precisely those that have been observed and in this case, $\beta _{2}^{(0)}=0.91$ was obtained.

We will assume that the correlation between the marginal distributions of fatal and non-fatal time to events is weak (*ρ*=0.1). Table 2 shows the assumed parameters, together with the number of events and sample size required, the probability of observing the composite event and the empirical power.

**Table 2:** Summary of the scenario considered for the ARREST trial based on estimations from the raw data

*β*_1_	*β*_2_	*ρ*	gAHR(12)	Events	${p_{*}^{(a)}}$	N	Emp. Power
0.70	0.91	0.1	0.788	555	0.171	3,238	0.805

***gAHR***(12): geometric Average Hazard Ratio; $\boldsymbol {p}_{*}^{\boldsymbol {(a)} }$: Probability of observing composite event; ***N***: Sample size rounded to an even number

Some meaningful conclusions emerge from Table 2. First of all, the number of patients required taking into account an expected 10% follow-up losses would be 3,598 (=3,238/0.90) patients. This is a clearly higher number of patients than planned in the ARREST trial (*n*=770). This is mainly due to the fact that the ARREST trial protocol anticipated a much greater treatment effect on OS (going from a proportion of 16% to 9% is equivalent to a relative risk of 0.56) when in fact it resulted in a *HR*_*OS*_=1.10, and this why we are considering HR_1_=0.95 for OS. This almost negligible effect on OS together with the highest probability of observing OS causes the treatment effect on the other component to be blurred in the composite endpoint and results in a huge sample size. In this sense, a trial that deals with the most relevant component (bacteriologically confirmed treatment failure or disease recurrence) as primary endpoint would only require observing 29 events for a HR=0.35 and, assuming that mortality is independent of treatment failure or disease recurrence and that the event was observed in a proportion of patients equal to 0.05, it would involve 570 patients. Regarding the main objective of our work, the empirical power considerably trap the target power of 0.80.

**Fig. 2:**
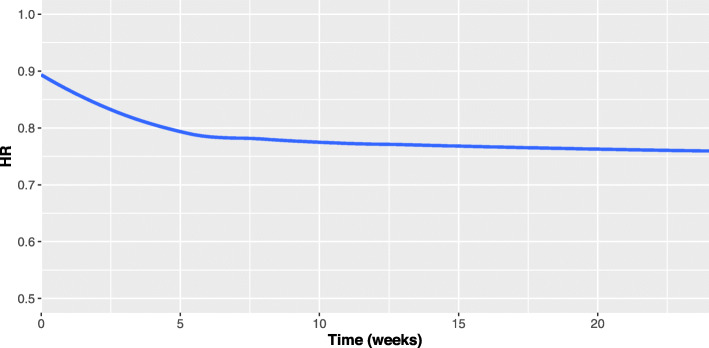
ARREST trial. HR_∗_(*t*) over time in the assumed scenario of the ARREST trial

CompARE can be used to plot the *HR*_∗_(*t*) for the considered scenario. Figure 2 represents the all-cause HR over time based on estimated parameters from the raw data. It is reasonably constant with a value slightly around to 0.80 for almost all the follow-up except for the earlier times. So, a summary measure such as gAHR could serve to describe the hazard ratio and its estimated value gAHR=0.788 would be used in the design stage of a new study to calculate the needed sample size.

### Simulation studies

#### Simulation settings

The aim of the simulation study is to evaluate if the proposed method for calculating the sample size reaches the desired empirical power under different scenarios. Furthermore, we compare the proposed method to the naïve method resulting from averaging the HRs of the components. This measure would be very likely the choice of a trialist in the absence of further information. From now on, we will call this new measure the naive HR (nHR) and the method associated the naive method.

We have chosen scenarios that represent realistic situations when designing an RCT [11]. The probabilities $p_{1}^{(0)}$ and $p_{2}^{(0)}$ of observing each event in the control group have been taken between 0.1 and 0.5; the cause-specific hazard ratios *HR*_1_ and *HR*_2_ of each component from 0.6 to 0.9; the times until the events for each component (${\mathcal {E}}_{k}, \ k=1,2)$ have been modeled according to Weibull distributions with constant (*β*_*k*_=1), decreasing (*β*_*k*_=0.5) or increasing hazards (*β*_*k*_=2); and the correlations between these times have been selected from low to moderately-high (from *ρ*=0.1 to *ρ*=0.5). In addition, three different copulas (Frank, Clayton and Gumbel) were implemented to model the joint distribution. For simplicity, and without loss of generality, we have scaled the problem to a unit time of follow-up (*τ*=1).

We have considered two different settings. Setting 1 (405 scenarios) assumes that the times $\left (T_{1}^{(0)}, T_{2}^{(0)}\right)$ are exponentially distributed, while Setting 2 (3,240 scenarios) considers $\left (T_{1}^{(0)}, T_{2}^{(0)}\right)$ Weibull distributed. Scenarios with observed proportions $p_{1}^{(0)}=p_{2}^{(0)}=0.5$ are not realistic since they represent scenarios without censoring and have not been considered (See Table 3). Frank’s copula has been chosen to bind $(T_{1}^{(0)}, T_{2}^{(0)})$ in all the scenarios of both settings to compute the empirical powers. In order to assess the relevance of the copula’s choice on the results, the *gAHR* has been calculated in all the scenarios of both settings and through 3 different copulas (Frank, Clayton and Gumbel).

**Table 3:** Input parameters considered in the simulation according to the setting

	$p_{1}^{(0)},p_{2}^{(0)}$				**HR**_1_,HR_2_			*ρ*			*β*_1_,*β*_2_			Copula	Scenarios
	0.05	0.1	0.3	0.5	0.6	0.8	0.9	0.1	0.3	0.5	0.5	1	2	Frank	
Setting 1	x	x	x	x	x	x	x	x	x	x		x		x	396
Setting 2	x	x	x	x	x	x	x	x	x	x	x	x	x	x	3,168

Lows the total number of simulated scenarios. Scenarios with observed proportions $p_{1}^{(0)}=p_{2}^{(0)}=0.5$ in both settings as well as scenarios with exponential distribution in both components in Setting 2 are not considered

#### Simulation procedure

We ran 10,000 iterations for each scenario described in Table 3.

The empirical power of one-sided logrank test for the composite endpoint is computed using a statistical significance of *α*=0.025. Given Frank’s copula and a set of input parameters ($\beta _{1}, \beta _{2}, p_{1}^{(0)}, p_{1}^{(1)}, HR_{1}, HR_{2}, \rho $), the simulation was conducted following the next steps:

- **Parameter of the copula**. The copula parameter, *θ*, which defines the association between both components is calculated from the Spearman’s correlation coefficient, *ρ*. The relationship between these parameters is one-to-one and given in (13).
- **Scale parameters**. Based on Eqs. 14 and (15), we can numerically obtain the scale parameters of the Weibull marginal distribution in groups 0 and 1, respectively using the *multiroot* function of the *rootSolve* R package.
- **Geometric average hazard ratio,*gAHR(τ)***. *gAHR*(*τ*) is calculated following Eq. 2, which depends on $HR_{*}(t)=\lambda _{*}^{(1)}(t)/\lambda _{*}^{(0)}(t)$. *HR*_∗_(*t*) was numerically estimated for 1,000 equidistant points over the follow-up time (from 0 to *τ*).
- **Sample size**. For a given power of 1−*β*=0.8 and one-sided significance level *α*=0.025, together with the *gAHR*(*τ*), Eq. 11 is used to compute the sample size.
- **Generate data**. For each of the 10,000 iterations, bivariate data with the sample size obtained in the previous step was generated via Frank’s copula and using the *Mvdc* function of the *copula* R package. The simulated data was censored at the end of follow-up and takes into account the competing risks.
- **Test**. For each of the 10,000 iterations, the longrank test is conducted on the data and the statistic *Z*_∗_ for the composite endpoint *T*_∗_ is stored. This test is implemented in the *survdiff* function of the *survival* R package.
- **Empirical power**. The empirical power is estimated as the proportion of *Z*_∗_ statistics falling into the rejection region, i.e, *Z*_∗_<−1.96 along all the iterations.

All simulations were performed using the R version 3.6.1. We have not run the simulation for those scenarios with an associated sample size greater than 20,000 (both groups) due to their computational cost and because they do not represent realistic setups in the scope of RCTs. The R code for simulations, not supported by CompARE, is available at https://github.com/jordicortes40/sample_size_composite.

#### Simulation results for exponential case

**Fig. 3:**
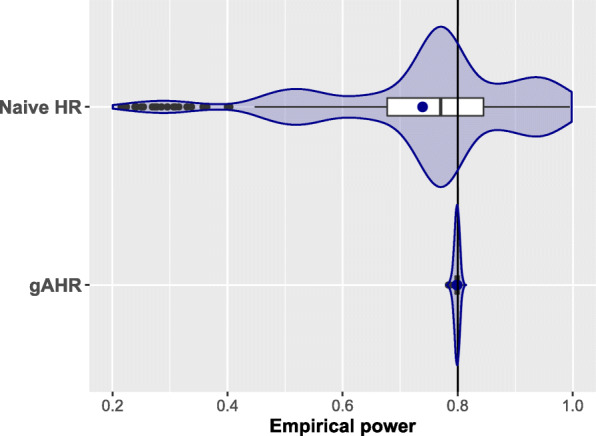
Exponential scenarios. Empirical power for the exponential case

From the 405 scenarios of this setting, we have excluded 9 (2.2%) and 3 scenarios (0.74%) with a sample size larger than 20,000 (both groups) when using the gAHR and nHR, respectively. These cases correspond to scenarios where both HRs are equal to 0.9 and the observed proportions of the events are equal or less than 0.10. We summarize in Fig. 3 and Table 4 the empirical powers for both measures (gAHR and nHR) corresponding to Setting 1 where $T_{1}^{(0)}$ and $T_{2}^{(0)}$ are exponentially distributed and bound via Frank’s copula.

**Table 4:** Empirical power according to the different input parameters in setting 1 (exponential case) using both measures (gAHR/nHR)

		Power descriptive				
	s	Min	Q1	Med	Q3	Max
Treatment effect						
Any *HR*_*j*_=0.6	225/225	0.784/0.215	0.795/0.601	0.798/0.765	0.801/0.882	0.809/0.995
*HR*_1_=*HR*_2_=0.9	36/42	0.789/0.727	0.798/0.762	0.801/0.775	0.803/0.786	0.808/0.799
Other cases	135/135	0.790/0.466	0.797/0.696	0.800/0.771	0.802/0.836	0.811/0.952
Observed proportions						
Any $p_{j}^{(0)}=0.05$	180/186	0.784/0.215	0.795/0.650	0.798/0.777	0.801/0.867	0.809/0.995
$p_{1}^{(0)}=p_{2}^{(0)} \ge 0.3$	81/81	0.790/0.536	0.797/0.708	0.800/0.764	0.803/0.791	0.810/0.901
Other cases	135/135	0.788/0.274	0.796/0.612	0.799/0.764	0.802/0.877	0.811/0.986
Correlation						
Weak (*ρ*=0.1)	132/134	0.788/0.251	0.796/0.698	0.798/0.789	0.802/0.855	0.811/0.995
Mild (*ρ*=0.3)	132/134	0.785/0.239	0.796/0.671	0.799/0.767	0.802/0.844	0.809/0.994
Moderate (*ρ*=0.5)	132/134	0.784/0.215	0.796/0.654	0.799/0.751	0.802/0.831	0.810/0.993
Global	396/402	0.784/0.215	0.796/0.678	0.799/0.771	0.802/0.845	0.811/0.995

First column (*s*) is the number of scenarios. *Min*: minimum; *Q1*: first quartile; *Med*: Median; *Q3*: third quartile; *Max*: maximum

In the 396 included scenarios using the gAHR, the required number of events (equation 12) ranges from 122 to 3,338 with a median equal to 644 [IQR: 222-1,254] and the total sample size ranges from 176 to 17,402 with a median equal to 1,644 [IQR: 600-4,157].

Figure 3 shows a violin-boxplot comparing the empirical powers achieved with gAHR and nHR methods, respectively merging all scenarios. For the former, both mean (solid point) and median are equal to 0.799, very close to the target power 0.80 taking into account the simulation mean standard error of 0.004. Moreover, this violin-boxplot shows an almost perfect symmetry with respect to the mean and the median, indicating a similar propensity to move away from the central tendency towards both higher and lower values. On the contrary, the naive method provides empirical powers with a mean/median lower than desired (0.771/0.739) and involves powers in a wide range from 0.22 to 0.99.

Table 4 presents summary statistics for the empirical power from both methods according to different categories for the input parameters: 1) Stratifying by treatment effect: i) one of the two components has a large treatment effect (*HR*_*k*_=0.6,*k*=1 or 2); ii) both components have a small treatment effect (*HR*_*k*_=0.9,*k*=1,2); iii) the remainder cases; 2) Stratifying by the probability of observing the event in the control group: i) at least one of the two components has a very small probability ($p_{k}^{(0)}=0.05, k=1\ \text {or} \ 2$); ii) both components have equal and large probabilities of being observed ($p_{1}^{(0)}=p_{1}^{(0)}\geq 0.3$); iii) the remainder cases; 3) Considering three different values for the correlation between both endpoints.

Overall, using the gAHR, 95.5% of scenarios had an empirical power between 0.79 and 0.81 in front of only 6.5% scenarios in the same interval using the nHR. The results based on the gAHR method were quite consistent among all strata. The first quartile of empirical power was, at least, 0.795 for any considered stratum, indicating that a power of no more than half a percentage point less than the target power will be achieved in 75% of the situations. There were 16 scenarios (4.0%) with empirical power below 0.790. This percentage was slightly higher when a large treatment effects (*HR*_*k*_=0.6) was present in any of the components (6.2%) or if at least one of the observed proportions was equal to 0.05 (7.2%). Both scenarios had lower treatment effects (*HR*_*k*_≥0.8) and observed proportions in the control group between 0.1 and 0.3 for both components. It is worth mentioning that no relevant differences were observed in the empirical power according to the correlation, but it should be borne in mind that for high correlations, a larger sample size was required to achieve the same power. The set of scenarios where the application of the naive method would lead to fairly good control of power would be those with very similar treatment effect on both components. For example, as can be seen in the table, when both HRs are equal to 0.9, there may be a decrease in the desired power – due to the competing events – but it would not go beyond around 7% in the worst situation.

#### Simulation results for non-exponential cases

**Fig. 4:**
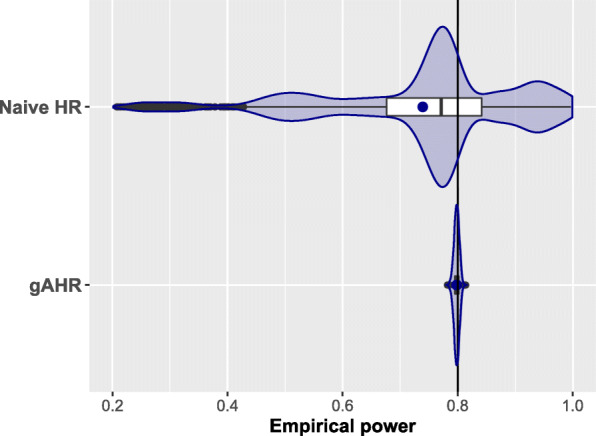
Non-exponential scenarios. Empirical power for the non-exponential cases

From the 3,240 scenarios considered in the setting 2, we excluded, as in the exponential setting 1, 72 scenarios (2.2%) using the gAHR and 24 scenarios (0.74%) using the nAHR with a sample size larger than 20,000. Again, HRs equal to 0.9 and probabilities in the control arm equal or less than 0.10 provided these situations that required extreme sample sizes. We summarize in Fig. 4 and Table 5 the results of the remaining scenarios of setting 2 where $T_{1}^{(0)}$ and $T_{2}^{(0)}$ are bound via Frank’s copula and Weibull distributed with shape parameters *β*_1_ and *β*_2_ equal to 0.5,1,2, excluding the case in which both are exponential (*β*_1_=*β*_2_=1). Thus, we included extreme scenarios where the hazard trends over time of both components pointed out in opposite directions with increasing (*β*_*k*_=2) and decreasing (*β*_*k*_=0.5) hazard rates in one and the other component, respectively.

**Table 5:** Empirical power according to scenarios in setting 2 (non-exponential case) using both measures (gAHR/nHR)

		Power descriptive				
	s	Min	Q1	Med	Q3	Max
Treatment effect						
Any *HR*_*j*_=0.6	1800/1800	0.782/0.208	0.794/0.596	0.797/0.770	0.801/0.884	0.813/0.997
*HR*_1_=*HR*_2_=0.9	288/336	0.789/0.724	0.797/0.762	0.800/0.774	0.803/0.786	0.810/0.804
Other cases	1080/1080	0.787/0.457	0.796/0.692	0.799/0.772	0.802/0.835	0.812/0.957
Observed proportions						
Any $p_{j}^{(0)}=0.05$	1440/1488	0.782/0.208	0.794/0.645	0.797/0.777	0.801/0.870	0.813/0.997
$p_{1}^{(0)}=p_{2}^{(0)} \ge 0.3$	648/648	0.788/0.420	0.797/0.717	0.800/0.768	0.802/0.790	0.813/0.949
Other cases	1080/1080	0.783/0.250	0.796/0.609	0.798/0.766	0.801/0.878	0.811/0.991
Correlation						
Weak (*ρ*=0.1)	1056/1072	0.783/0.252	0.795/0.693	0.799/0.789	0.801/0.859	0.811/0.995
Mild (*ρ*=0.3)	1056/1072	0.783/0.226	0.795/0.671	0.798/0.770	0.801/0.841	0.813/0.996
Moderate (*ρ*=0.5)	1056/1072	0.782/0.208	0.795/0.645	0.798/0.753	0.801/0.828	0.812/0.997
Laws of the components						
*β*_1_=*β*_2_=0.5	396/402	0.786/0.223	0.796/0.676	0.798/0.771	0.801/0.844	0.812/0.995
*β*_1_=*β*_2_=2	396/402	0.783/0.219	0.796/0.680	0.799/0.770	0.801/0.842	0.811/0.995
*β*_1_≠*β*_2_	2376/2412	0.782/0.208	0.795/0.677	0.798/0.772	0.801/0.840	0.813/0.997
Global	3168/3216	0.782/0.208	0.795/0.677	0.798/0.771	0.801/0.841	0.813/0.997

First column (*s*) is the number of scenarios. *Min*: minimum; *Q1*: first quartile; *Med*: Median; *Q3*: third quartile; *Max*: maximum

In the included 3,168 scenarios with calculations founded on the gAHR, the needed number of events ranges from 122 to 3,356 with a median equal to 642 and the total sample size ranges from 176 to 17,402 with a median equal to 1,616. Figure 4 shows the empirical power for the 10,000 simulations. Again, both the mean and the median (0.798) are quite close to 0.80 and the violin-boxplot reveals a symmetry regarding to these statistics. Meanwhile, the naive method does not get a suitable power control and provides powers that on average (0.739) are lower than the target value, even with extreme scenarios giving probabilities to detect the treatment effect as low as 0.208 or as high as 0.997.

Table 5 presents summary descriptive statistics of the empirical power according to the same categories we defined for the exponential case. We have included a fourth strata splitting the empirical power according to equal and decreasing hazard rates (*β*_1_=*β*_2_=0.5); equal and increasing hazard rates (*β*_1_=*β*_2_=2) and the remaining scenarios including different hazard behaviour over time (*β*_1_≠*β*_2_).

Empirical power derived from the *gAHR* ranged from 0.782 to 0.813 and 95.7% of the scenarios were between 0.79 and 0.81, while only 6.9% scenarios remains within this range for the naive method. Regarding the results coming from the gAHR, the first quartile of empirical power was, at least, 0.794 in any strata and 0.795 overall, which guarantees a power that at most will be only half a percentage point lower than the target in 75% of scenarios. Almost all situations (99.7%) reflecting lower treatment effects (*HR*_*k*_=0.9) provided an empirical power above 0.79. There were 135 (4.0%) scenarios with empirical power below 0.790. This percentage was higher when a marked treatment effect (*HR*_*k*_=0.6) was present in any of the components (6.3%) or when at least one of the proportion of observed events in the control group was equal to 0.05 (5.8%). The 10 (0.3%) scenarios that presented an empirical power slightly higher than 0.81 did not share any common feature regarding to the input parameters and it could be explained due to the standard error ($\sqrt {\frac {0.8 \times 0.2}{10,000}}=0.004$) associated to the simulation procedure. Different levels of correlation between components did not provide relevant discrepancies. The conclusions for the naive method are the same in this non-exponential setting as in the exponential setting

#### Effect of copula on **gAHR**

The *gAHR* was calculated in all scenarios of settings 1 and 2 and for the 3 Archimedean copulas (Frank, Clayton and Gumbell) in order to assess the relevance of the copula binding $T_{1}^{(0)}$ and $T_{2}^{(0)}$. Table 6 provides the deciles of the *gAHR* values for each copula.

**Table 6:** Percentiles of the *gAHR* according to copula

	10%	20%	30%	40%	50%	60%	70%	80%	90%
Frank	0.62	0.66	0.71	0.76	0.80	0.81	0.84	0.87	0.90
Clayton	0.60	0.65	0.69	0.73	0.76	0.79	0.82	0.85	0.88
Gumbel	0.60	0.65	0.70	0.76	0.80	0.81	0.84	0.87	0.90

Overall, the deciles of the *gAHR* values obtained from either one of the 3 copulas are very similar. In particular, Frank’s and Gumbel’s Copula show identical values in *gAHR* percentiles from the 40% percentile on while Clayton’s copula slightly differs from both. The maximum absolute difference among any two copulas, found around the *gAHR* median, is 0.04, and, although small, it might have an important impact on the computation of the required number of events. For instance, going from a *gAHR*=0.80 to *gAHR*=0.76 implies a 34% decrease in the number of required events, as it happens when using Schoenfeld’s formula and the HR. Based on these simula- tion findings and others not shown here, we recommend the use of Frank’s copula to bind the joint distribution of the components unless more information can be gathered on the real correlation and the joint behaviour between both components. Nevertheless, CompARE allows the use of these 3 copulas, among others, which can be useful to calculate the HR_∗_(*t*) under different association patterns (e.g., stronger correlations at earlier or later times) between the component times.

## Discussion

We have shown that the use of gAHR in conventional sample size formulas in time-to-event studies with composite time-to-event endpoints provides the desired power when two treatment groups are compared using the log-rank test. This is true regardless of whether the proportional hazards assumption holds or not. In studies involving a composite endpoint, obtaining the theoretical value of this summary measure could be hard; however, CompARE has proven to be a useful tool for this purpose, removing tedious calculations. The gAHR method enhances the performance over the rule of thumb approach based on averaging the treatment effects on each component.

**Fig. 5:**
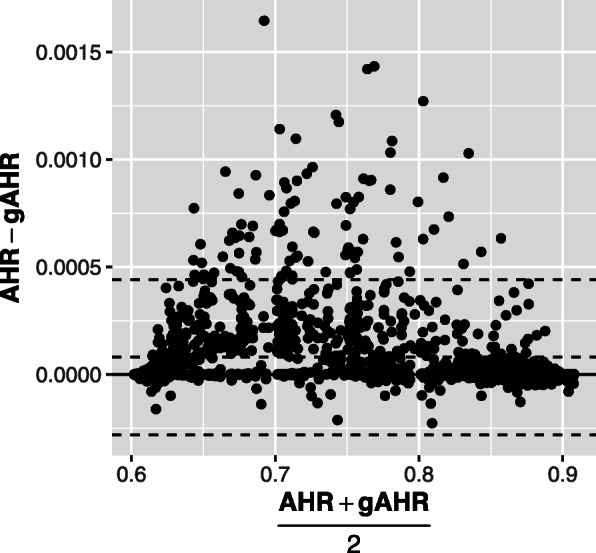
gAHR versus AHR. Relationship between gAHR and AHR in the scenarios of settings 1 and 2

The use of the HR has been debated when hazards are not proportional. Also, the hazard ratio as estimated from the observed data doesn’t reflect the causal hazard ratio if there is heterogeneity in patient risk [19, 20]. Other measures such as the restricted mean survival time may be better to quantify treatment effects. But, actually, the HR is still widely used and most of the published RCTs report the HR as the main measure of the treatment effect. Furthermore, the estimand exp(*β*) itself still has an interpretation as the ratio of the logarithms of the survival functions log[*P*(*T*^(1)^>*t*)]/ log[*P*(*T*^(0)^>*t*)]. In this sense, several summary measures have been proposed to capture the treatment effect when the HR_∗_(*t*) varies considerably over time. Probably the most popular is the *AHR* proposed by Kalbfleisch and Prentice [5], which is mentioned in several studies as a good measure of the effect of an intervention [6, 7]. The gAHR, in most of our simulation settings, presents values very close to the AHR as shown in the Bland-Altman plot of concordance (Fig. 5). This point is interesting from the perspective that *gAHR* could be interpreted as a measure of proportional hazards just in the same way as *AHR* and, in addition, the former has the advantage of a direct relationship with the sample size calculation.

One question that may arise is how sensitive the sample size and power are to misspecification of some of the input parameters. Of course, a misspecified HR will always influence the needed number of events and consequently, the power in any sample size calculation. Some information regarding the proportion of events observed is also essential to deduce the number of patients from the number of events. Our methodology requires additional inputs, such as the shape parameters of the Weibull distributions; the degree of association among the times to event of both components; or the choice of the copula. They are mostly nuisance parameters, so ideally the sample size would be quite insensitive to the assumptions that is made. We have seen that they are not very sensitive to the choice of copula. But the shape parameters of the Weibull distribution or the correlation might influence the resulting sample size if we blunder in the choice. For the former, the users should draw on their experience to determine the direction of risk over time by assessing whether the event rate increases, decreases or remains constant during the follow-up. For example, an outcome variable about the infection after a surgery, the most critical stage is just after the intervention and subsequently, the risk decreases over time. Regarding the correlation, obviously, it cannot be determined with certainty, but a rule of thumb would be enough to approximate it. For instance, events that rarely occur in the same patient should be weakly associated or, conversely, if the events are usually dependent on the patient’s characteristics, then it can lead to a moderate/high correlations.

Our investigation has several limitations. First, we have only addressed the case of a composite endpoint of two components. In scenarios with more than two possible outcomes, we recommend to combine them first into two groups according to their relevance or their expected effectiveness as long as it is feasible to anticipate the parameters associated with these components based on previous literature or previous RCT phases. Second, we are aware that the latent failure time model that we are imposing has been criticised because the dependence structure between $T_{1}^{(g)}$ and $T_{2}^{(g)}$ is in general not identifiable since the latent times are not observable. Nevertheless, latent times are the predominant approach for simulating competing risks data as it is discussed by Allignol et al. [21] as long as they yield the right data structure and they are computationally correct. We remind as well here that we are at the design phase of the RCT and that we are not addressing the estimation of the treatment effect measures. Third, since we are in a competing risk situation, the hazard of having one of the event types is influenced by the other competing event, making for a complex interference. If, in addition, treatment effects are different on both events, the proper definition of the “at risk”-sets is involved and the intuition for what “proportional cause-specific hazards” means is not straightforward. However, this is the information that can be usually extracted from previous published studies and, therefore, the one that can be used to estimate the sample size for future trials. Fourth, we have only dealt with the situation of equal-sized treatment groups. Although it is well known that the situation that maximizes the power is the one in which the events are balanced among groups, RCTs usually are designed to balance the number of patients among the different treatment arms.

## Conclusions

The waste associated with biomedical research is paramount [22] and one of the main problems is poorly designed studies. Underestimating the optimal sample size may lead to failure to make effective interventions available to patients due to unsuccessful trials. On the other hand, trials with too many patients might unnecessarily subject people to ineffective interventions. One may use CompARE platform (https://cinna.upc.edu/CompARETimeToEvent/) to design randomized controlled trials with composite endpoints when the proportional hazards assumption does not hold.

## Data Availability

The only dataset analysed during the current study comes from the ARREST trial. The data are not publicly available because the authors are not the owners of the data and they do not have the permission to made them publicly available. Data are however available from the authors upon reasonable request and with permission of the owner of the data MRC Clinical Trials Unit of UCL (https://www.ctu.mrc.ac.uk/), or can be asked for directly from the owner. Declarations
